# Clinical Features of Patients with Home Isolation Sars-Cov-2 Infection: A Multicenter Retrospective Study in Southern Italy

**DOI:** 10.3390/life11040347

**Published:** 2021-04-16

**Authors:** Mariantonietta Pisaturo, Giulia De Angelis, Paolo Maggi, Vincenzo Sangiovanni, Fabio Giuliano Numis, Ivan Gentile, Alfonso Masullo, Carolina Rescigno, Giosuele Calabria, Angelo Salomone Megna, Michele Gambardella, Elio Manzillo, Giancarlo Giolitto, Annamaria Rossomando, Antonio Riccardo Buonomo, Margherita Macera, Vincenzo Messina, Antonio Pagano, Raffaella Pisapia, Nunzia Farella, Giorgio Bosso, Nicola Coppola, CoviCam Group

**Affiliations:** 1 Infectious Diseases, Department of Mental Health and Public Medicine, University of Campania “L. Vanvitelli”, 80100 Naples, Italy; mariantonietta.pisaturo@unicampania.it (M.P.); giulia.e19@gmail.com (G.D.A.); macera.margherita@libero.it (M.M.); 2 Third Infectious Diseases Unit, AORN dei Colli, P.O. Cotugno, 34105 Naples, Italy; sangio.vincenzo@gmail.com; 3 Infectious Diseases Unit, A.O. S Anna e S Sebastiano, 23868 Caserta, Italy; paolo.maggi@unicampania.it (P.M.); vincenzomessina.ce@libero.it (V.M.); 4 Emergency Unit, PO Santa Maria delle Grazie, 80078 Pozzuoli, Italy; fabiogiuliano.numis@aslnapoli2nord.it (F.G.N.); antonio.pagano@aslnapoli2nord.it (A.P.); giorgio.bosso@aslnapoli2nord.it (G.B.); 5 Infectious Disease Unit, University Federico II, 34105 Naples, Italy; ivan.gentile@unina.it (I.G.); antonioriccardobuonomo@gmail.com (A.R.B.); 6 Infectious Disease Unit, A.O. San Giovanni di Dio e Ruggi D’Aragona, 84100 Salerno, Italy; al.masullo@alice.it; 7 First Infectious Disease Unit, AORN dei Coli, PO Cotugno, 34105 Naples, Italy; carolinarescigno@libero.it (C.R.); raffipisapia@gmail.com (R.P.); 8 IX Infectious Disease Unit, AORN dei Coli, PO Cotugno, 34105 Naples, Italy; g.calabria@tin.it (G.C.); nunzia.farella@libero.it (N.F.); 9 Infectious Disease Unit, A.O. San Pio, PO Rummo, 82100 Benevento, Italy; angelo.salomonemegna@ao-rummo.it; 10 Infectious Disease Unit, PO S. Luca, Vallo della Lucania, ASL, 84100 Salerno, Italy; gambardella1960@gmail.com; 11 VIII Infectious Disease Unit, AORN dei Coli, PO Cotugno, 34105 Naples, Italy; manzillo@libero.it; 12 Infectious Disease Unit, Ospedale Maria S.S. Addolorata di Eboli, ASL, 84100 Salerno, Italy; giancarlo.g@libero.it; 13 IV Infectious Disease Unit, AORN dei Coli, PO Cotugno, 34105 Naples, Italy; annamaria.rossomando@gmail.com; 14 Department of Mental Health and Public Medicine, Section of Infectious Diseases, University of Campania Luigi Vanvitelli, Naples, Via L. Armanni 5, 80131 Naples, Italy

**Keywords:** COVID-19, SARS-CoV-2 infection, mild clinical presentation, home management, home isolation

## Abstract

To describe epidemiological and clinical features of patients confirmed as having SARS-CoV-2 infection and managed in isolation at home. We performed a multicenter retrospective study enrolling all SARS-CoV-2-positive adults evaluated from 28 February to 31 May 2020 at one of nine COVID-19 Units in southern Italy: we included patients receiving care at home and those admitted to hospital. We defined patients with not-severe disease if they were asymptomatic or experienced a mild infection that did not need oxygen (O_2_) therapy and those with a severe infection if hospitalized and required O_2_ therapy. We enrolled 415 patients with SARS-CoV-2 infection: 77 were managed in isolation at home, 338 required hospital management. The 77 patients in home isolation were less frequently male than hospitalized patients (55% vs. 64%; <0.01) and were younger (median age 45 years (IQR:19) vs. 62 (IQR 22); *p* < 0.01), had a lower Charlson comorbidity index (median 0 (IQR2) vs. 6 (IQR 3); *p* < 0.01), and included fewer subjects with an underlying chronic disease (36% vs. 59%; *p* < 0.01). According to a binomial logistic regression analysis, a younger age (OR: 0.96 (95% IC: 0.94–0.98), *p* < 0.01) and a low Charlson comorbidity index (OR: 0.66 (95% IC: 0.54–0.83); *p* < 0.01) were independent factors associated with at-home management. The identification of subjects with SARS-CoV-2 infection who could be managed in home isolation is useful in clinical practice. A younger age and no comorbidities were identified as factors independently associated with home management.

## 1. Introduction

Since December 2019, a new zoonotic beta-corona virus, the severe acute respiratory syndrome coronavirus-2 (SARS-CoV-2), has spread all over the world, causing a disease known as Coronavirus disease-19 (Covid-19) [1,2,3].

This infection is transmitted from human-to-human through nasal or oral droplets or through close contact; fecal–oral transmission has a modest epidemiologic impact [2,4,5,6]. The infection showed a mean incubation period of 5.2 days (range 2–14) [7,8]; COVID-19 infection has a broad spectrum of severity ranging from an asymptomatic form to severe acute respiratory syndrome, which requires mechanical ventilation. The early presentation of COVID-19 infection is typically non-specific. Among symptomatic patients, about 80% showed a mild clinical course [1,2,3,9,10,11] characterized by a dry cough, sore throat, low-grade fever, or malaise; these patients should be managed in isolation at home. However, in 20% of symptomatic cases, the clinical condition worsened in about 7 days from the beginning of the symptoms, especially respiratory failure [3,12,13]. Little data are available in the literature on the clinical features characteristic of patients who could be managed at home, so clinical criteria and biomarkers are needed to help differentiate between individuals more likely to progress to a severe illness. 

We described the epidemiological and clinical characteristics of patients confirmed as having SARS-CoV-2 infection who were managed at home due to pauci-symptomatic forms of SARS-CoV-2 infection and compared the features of these patients with those hospitalized.

## 2. Methods

### 2.1. Study Design and Setting

We performed a multicenter, observational cohort study involving nine COVID-19 Units in seven cities in the Campania region in southern Italy: Naples, Caserta, Salerno, Benevento, Pozzuoli, Eboli and Vallo della Lucania.

The study population included all adult patients (≥18 years) with a diagnosis of SARS-CoV-2 infection confirmed by a positive reverse transcriptase-polymerase chain reaction (RT-PCR) on a naso-oropharyngeal swab, symptomatic or asymptomatic, evaluated at one of the centers participating in the study from 28 February to 31 May 2020. We included patients admitted to hospital and those receiving care at home; in the latter case, the patients were evaluated by video calls. No study protocol, or guidelines regarding criteria of hospitalization and treatment recommendations were shared among the centers involved in this study; however, all the centers followed the regional guidelines on the management of the patients with COVID-19 [14]. In the Campania region, all the SARS-CoV-2 patients were managed at home unless they had an underlying disease or they showed signs of respiratory injury, regardless of their socio-economic status or other factors. Antiviral treatments were begun according to the decision of the physicians of each center. Exclusion criteria included minority age, the non-availability of clinical data and the absence of informed consent.

All demographic, clinical, laboratory and radiological data and therapy details of both hospitalized and non-hospitalized patients were collected in a database we created at the end of February 2020, when the infection by SARS-CoV-2 started to spread in our country. 

The study was approved by the Ethics Committee of the University of Campania L. Vanvitelli, Naples (n°10877/2020). All procedures performed in this study were in accordance with the ethical standards of the institutional and/or national research committee and with the 1964 Helsinki declaration and its later amendments, or comparable ethics standards. Informed consent was obtained from all participants included in the study.

### 2.2. Variables and Definitions

Microbiological diagnosis of SARS-CoV-2 infection was defined as a positive RT-PCR test on the naso-oropharyngeal swab.

We defined patients with not-severe SARS-CoV-2 infection if they were asymptomatic or experienced a mild infection and did not need oxygen (O_2_) therapy; we defined patients with a severe disease if they were hospitalized and required O_2_ therapy; in this definition we also included patients who died.

### 2.3. Statistical Analysis

For the descriptive analysis, categorical variables were presented as absolute numbers and their relative frequencies. Continuous variables were summarized as mean and standard deviation if normally distributed or as the median and interquartile range (IQR) if not normally distributed. 

All the variables with a *p* ≤ 0.05 at the univariate analysis and which were complete in all subjects enrolled in this study were entered in a regression logistic binomial analysis to identify factors independently associated with home isolation. Odds ratios (OR) with 95% confidence intervals (CI) were estimated using a binomial logistic regression model. To analyze the impact of comorbidities, in the logistic regression model we used Charlson Index Score as a categorical variable (<2 or ≥2 points), since the median of Charlson Index Score in the population enrolled was 2 points.

A *p*-value below 0.05 was considered statistically significant. Analyses were performed using SPSS 21.0 (IBM, Armonk, NY, USA).

## 3. Results

Four hundred and fifteen patients with COVID 19 disease were enrolled during the first wave of the SARS CoV2 epidemic in Italy in one of the 9 COVID 19 centers participating in the study.

The demographic and clinical characteristics of the 415 patients are shown in Table 1. Patients were predominantly males (62%), with a median age of 60 years (interquartile range, IQR, 24) (Table 1). The patients had a Charlson comorbidity index median of 2 (IQR, 0), with more than half of the patients with an underlying chronic disease, in particular arterial hypertension (40%), cardio-vascular disease (21%), diabetes (15%) and chronic obstructive pulmonary disease (18%) (Table 1). Fever (75.9%) and cough (35%) were the most frequent symptoms. Of the 415 patients enrolled, 77 (19%) were managed at home due to an asymptomatic (13 subjects, 16.9%) or pauci-symptomatic disease (64 patients, 83.1%), and 338 (81%) were hospitalized; 59 (13%) patients died.

Table 2 shows the demographic and clinical characteristics of the patients in home isolation and those hospitalized. Compared to hospitalized patients, those in home isolation were less frequently male (55% vs. 64%; *p* < 0.01) and were younger (median age 45 years (IQR = 19) vs. 62 (IQR = 22; *p* < 0.01) (Table 2)). Most patients in the home isolation group were healthcare workers (50% vs. 25%; *p* < 0.01). As regards to the presence of co-pathologies, home isolation patients had a lower Charlson co-morbidity index than the hospitalized patients (median 0 (IQR = 2) vs. 6 (IQR = 3); *p* < 0.01), and less frequently had an underlying chronic disease (36% vs. 59%; *p* < 0.01).

Of the 77 patients in home isolation, 13 (17%) were asymptomatic and 64 (83%) pauci-symptomatic; no patient in this group had a severe clinical disease or died (Table 2). On the contrary, none of the 338 hospitalized patients were asymptomatic, 199 (60.7%) showed a non-severe disease and 129 (38.2%) severe COVID-19; for 10 patients it was not possible to establish the severity of the disease. Fifty-nine (15.9%) of the patients in this group died (Table 2).

According to a binomial logistic regression analysis, a younger age (OR: 0.96 (95% IC: 0.94–0.98), *p* < 0.01) and a low Charlson comorbidity index (OR: 0.66 (95% IC: 0.54–0.83); *p* < 0.01) were independent factors associated with home management of COVID-19 disease (Table 3).

Interestingly, although the data on the clinical presentation were not complete, the subjects in home isolation less frequently showed fever (43/76 (56%) vs. 171/208 (81%), *p* < 0.01) or dyspnea (3/76 (3.9%) vs. 83/210 (24%), *p* < 0.01) and more frequently hypo-ageusia (27/76 (35%) vs. 35/160 (21.6%), *p* = 0.02) (Table 2).

Supplementary Table S1 shows the demographic and clinical characteristics of the patients in home isolation according to the presence or absence of symptoms. No difference was observed between the two groups of patients (Supplementary Table S1).

In the hospitalized patients, those with a severe clinical presentation were older (median age 60 (IQR 22) vs. 68 (IQR 22); *p* < 0.01)) and more often had an underlying chronic disease (53% vs. 71%, *p* = 0.01) than those without and were less frequently healthcare workers (25% vs. 13%; *p* = 0.02) (Supplementary Table S2).

## 4. Discussion

The present study evaluated the demographic and clinical features of SARS-CoV-2 infection in 77 patients in home isolation in southern Italy, with, as a term of comparison, the patients hospitalized in nine COVID 19 units.

The patients in home isolation due to an asymptomatic or pauci-symptomatic infection were younger than hospitalized patients. This observation is in line with the data in the literature showing that older age was associated with severe forms of COVID-19 and thus to the forms requiring hospitalization. In this regard, Zhou F et al. [1], one of the first to give a detailed description of COVID-19 in Wuhan, China, in a retrospective, multicenter cohort study, showed that older age was associated with an increasing risk of hospital death (OR: 1.10, 95% CI 1.03–1.17, per year increase; *p* = 0.0043). Similar data by the China Medical Treatment Expert Group showing that patients with severe disease were older than those with non-severe disease by a median of 7 years (52.0 (IQR: 40.0–65.0) vs. 45.0 (IQR: 34.0–57.0)) [2].

Interestingly, the subjects in home isolation more frequently were healthcare workers, perhaps because they felt more confident about staying at home, thinking they would understand if their health deteriorated or because they more frequently identified the infection in an asymptomatic phase due to active surveillance in the hospital setting. Probably, they were able to maintain contact with medical colleagues who monitored them at a distance through video calls, than subjects who were not healthcare workers, regardless of their socio-economic conditions. Similar results were obtained from a study by Ayaz: in a cohort of 41 patients with COVID-19 followed in home isolation, 70.7% were healthcare workers [15].

In addition, SARS-CoV-2 positive patients in home isolation less frequently had a comorbidity. In fact, they had a lower Charlson comorbidity index than hospitalized patients and fewer underlying chronic diseases. The association between the presence of chronic diseases and severe forms of COVID-19 are known [1,2,16,17,18]. For example, Zhou [1] et al. showed that comorbidities were present in nearly half of hospitalized patients, with hypertension being the most common, followed by diabetes and coronary heart disease. Similarly, Guan [2] found that a coexisting illness was more common among patients with a severe disease than those with a non-severe disease (38.7% vs. 21%) and Wang D et al. [3] showed that patients treated in the ICU, compared with patients not treated in the ICU, were more likely to have underlying comorbidities (72.2% vs. 37.3%). However, when we consider the group of the 338 hospitalized patients in the present study, a severe clinical course was associated with older age and the presence of comorbidities.

The data on the clinical features of subjects with SARS-CoV-2 infection in home isolation are scanty in the literature. In this regard, the results of a cohort of diabetic patients observed in home isolation are interesting: they enrolled 32 patients with COVID-19 and with type 1 diabetes (25 in home isolation and 7 hospitalized): no statistically significant relationship was observed between the 2 groups of patients [19]. Another study was carried out on 95 veterans testing positive for COVID-19 in Milwaukee, Wisconsin: 15 required mechanical ventilation, 50 were hospitalized without the need for ventilation and 31 were discharged to home isolation. In this survey, patients with COVID-19 who required hospitalization with mechanical ventilation were more likely to have a history of hypertension and hyper-lipidemia than patients who were discharged to home quarantine (85.7% and 78.6% vs. 48.4% and 45.2%, respectively; *p*  <  0.05 for each) [20].

In relation to the clinical presentation, the subjects with home management less frequently had dyspnea, but more frequently had hypo-ageusia than hospitalized patients. A survey from a community facility designated for the isolation of patients without moderate-to-severe symptoms of COVID-19 in South Korea found that of 172 patients with mild COVID-19, one of the most common symptoms was hyposmia (61/172 (35.4%) [13].

Even considering the limitations of the present study, given that it was retrospective and that the sample of patients observed in home isolation was small, the data shown can be of help to give a clearer understanding of subjects with SARS-CoV-2 infection managed in home isolation. In fact, the identification of factors associated with mild forms of infection may be useful when considering the hospitalization criteria and good use of resources. However, further studies are needed to confirm these data.

## Figures and Tables

**Table 1:** Demographic and clinical characteristics of the 415 patients enrolled.

	415 Patients
N° (%) of males	259 (62%)
Age, years, median (IQR)	60 (24)
N° (%) of patients in different age classes, (years)	
18–29	21 (5%)
30–39	40 (10%)
40–49	62 (15%)
50–59	79 (19%)
60–69	98 (23%)
70–80	74 (18%)
>80	41 (10%)
Days of enrolment after onset of symptoms, Median (IQR)	6 (6.25)
N° (%) of healthcare workers	68/224 (30%)
N° (%) of subjects with contact with a confirmed or suspected COVID-19 case	129 (31%)
Charlson comorbidity index, median (IQR)	2 (0)
N° (%) of subjects with underlying chronic disease	230 (55%)
- With hypertension	164 (40%)
- With cardio-vascular disease	87 (21%)
- With diabetes	59 (15%)
- With malignancy	33 (8%)
- With chronic kidney disease	32 (8%)
- With chronic obstructive pulmonary disease	52 (13%)
- With liver cirrhosis	6 (2%)
N° (%) of asymptomatic subjects	13/405 (3.1%)
N° (%) of symptomatic subjects	392/405 (94.4%)
N° (%) of subjects in home isolation	77(19%)
N° (%) of hospitalized patients	338 (81%)

**Table 2:** Demographic and clinical characteristics of in-home isolation and hospitalized patients.

	In-Home Isolation	Hospitalized	*p*
N° of subjects	77	338	
N° (%) of males	42/77 (55%)	217/338 (64%)	<0.01
Age, years, median (IQR)	45 (19)	62 (22)	<0.01
N° (%) of patients in different age classes, (years)			
18–29	12 (16%)	9 (2%)	<0.01
30–39	14 (18%)	26 (7%)	<0.01
40–49	22 (29%)	40 (12%)	<0.01
50–59	13 (17%)	66 (20%)	0.23
60–69	14 (18%)	84 (25%)	0.21
70–80	1 (1%)	73 (22%)	<0.01
>80	1 (1%)	40 (12%)	<0.01
Days of enrolment after onset of symptoms, Median (range)	5	6	0.32
N° (%) of healthcare workers	24/48 (50%)	44/176 (25%)	<0.01
N° (%) of subjects with contact with suspected or confirmed COVID-19 case	30/54 (39%)	128/204 (38%)	0.27
Charlson comorbidity index, median (IQR)	0 (2)	6 (3)	<0.01
N° (%) of subjects with underlying chronic disease	28/75 (36%)	198/333 (59%)	<0.01
- With hypertension	21/75 (27%)	141/297 (42%)	<0.01
- With cardio-vascular disease	7/75 (9%)	79/307 (23%)	<0.01
- With diabetes	3/75 (4%)	55/309 (16%)	<0.01
- With malignancy	0/77	31/310 (9%)	<0.01
- With chronic kidney disease	3/75 (4%)	29/300 (9%)	0.12
- With chronic obstructive pulmonary disease	3/75(4%)	48/309 (14%)	<0.01
- With liver cirrhosis	0/77	6/274 (2%)	0.19
N° (%) of asymptomatic subjects	13/77 (17%)	0/338	<0.01
N° (%) of symptomatic patients	64/77 (83%)	338/338 (100%)	
N° (%) of symptomatic subjects with			
- fever	43/76 (56%)	171/208 (81%)	<0.01
- cough	35/76 (45%)	113/210 (33%)	0.25
- dyspnea	3/76 (4%)	83/210 (24%)	<0.01
- hypo ageusia	27/76 (35%)	35/160 (21.6%)	0.03
- hypo-anosmia	26/76 (34%)	73/169 (43.1%)	0.19
- diarrhea	6/76 (8%)	23/192 (7%)	0.33
- cutaneous lesions	2/76 (2%)	0/338	<0.01
Clinical presentation of COVID-19, N° (%)			
- non-severe COVID-19	77	199 (61) *	<0.01
- severe COVID-19	0	129 (39) *	<0.01
- death	0	59 (14%)	<0.01

* missing data for 10 subjects.

**Table 3:** Binomial logistic regression to identify factors independently associated with home isolation.

	*p* Value	OR	95% C.I.	
			Lower Limit	Upper Limit
Age	<0.01	0.96	0.94	0.98
Sex	0.72	0.89	0.49	1.64
Charlson Comorbidity Index	<0.01	0.66	0.54	0.82

C.I.: Confidence interval; OR: ODDS ratio.

## Data Availability

The data presented in this study are available on request from the corresponding author.

## Supplementary Materials

The following are available online at https://www.mdpi.com/article/10.3390/life11040347/s1, Table S1: Demographic and clinical characteristics of patients in home isolation according to the presence of symptoms, Table S2: Demographic and clinical characteristics of the hospitalized patients according to the COVID-19 severity.
