# Supplementary material for: Clinical Features of Patients with Home Isolation Sars-Cov-2 Infection: A Multicenter Retrospective Study in Southern Italy
Source: Life (Basel). 2021 Apr 16;11(4):347. doi: 10.3390/life11040347 (PMC8073533; doi:10.3390/life11040347)
Supplement: Supplementary file 1 [file life-11-00347-s001.zip › life-1153209-SI.pdf]

*Supplementary Materials*

# Clinical Features of Patients with Home Isolation Sars-Cov-2 Infection: A Multicenter Retrospective Study in Southern Italy

**Table S1.** Demographic and clinical characteristics of patients in home isolation according to the presence of symptoms.

| | Asymptomatic subjects | Symptomatic patients | P |
|---|---|---|---|
| **N° of subjects** | **13** | **64** | |
| **N° (%) of males** | 5 (38) | 37 (58) | .201436. |
| **Age, years, median (IQR)** | 46 (22.5) | 45 (18.75) | .933855. |
| **N° (%) of patients in different age classes, (years)** | | | |
| **18-29** | 2 (15%) | 10 (16) | .982619. |
| **30-39** | 2 (15%) | 12 (19) | .774251. |
| **40-49** | 4 (31%) | 19 (30) | .938075. |
| **50-59** | 4 (31% | 9 (14) | .142646. |
| **60-69** | 1 (8%) | 12 (19) | .331893. |
| **70-80** | 0 | 1 (1) | |
| **>80** | 0 | 1 (1) | |
| **Days of enrolment after onset of symptoms, median (range)** | // | 5 | |
| **N° (%) of healthcare workers** | 5 (39) | 24 (38) | .9003 |
| **N° (%) of subjects with contact with suspected or confirmed COVID-19 case** | 10 (77) | 20 (31) | .0021 |
| **Charlson comorbidity index, median (IQR)** | 1 | 0 (2) | .613598. |
| **N° (%) of subjects with underlying chronic disease** | 6 (46) | 24/62 (38) | .182117. |
| **With hypertension** | 2/13 (15) | 18/62 (28) | .311673. |
| **With cardio-vascular disease** | 1/13 (8) | 6/62 (9) | .822985. |
| **With diabetes** | 2/13 (15) | 1/62 (1) | .02123. |
| **With malignancy** | 0 | 0 | |
| **With chronic kidney disease** | 0 | 3/62 (5) | |
| **With chronic obstructive pulmonary disease** | 1/13 (8) | 2/62 (3) | .454941. |
| **With liver cirrhosis** | 0 | 0 | |
| **N° (%) of asymptomatic subjects** | 13 (100) | 0 | |
| **N° (%) of subjects with** | | | |
| **fever** | // | 43/63 (56) | |
| **cough** | // | 35/63 (45) | |
| **dyspnea** | // | 3/63 (4) | |
| **hypo ageusia** | // | 27/63 (35) | |
| **hypo-anosmia** | // | 26/63 (34) | |
| **diarrhea** | // | 6/63 (8) | |
| **cutaneous lesions** | // | 2/63 (2) | |

**Table S2.** Demographic and clinical characteristics of the hospitalized patients according to the COVID-19 severity.

| | Hospitalized patients | | p |
|---|---|---|---|
| | **Non-severe COVID-19** | **Severe COVID-19** | |
| **N° of subjects** | **196** | **132** | |
| **N° (%) of males** | 127 (65) | 86 (65) | .947229. |
| **Age, years, median (IQR)** | 60 (22) | 68 (22) | < .00001. |
| **N° (%) of patients in different age classes, (years)** | | | |
| **18-29** | 7 (4) | 2 (2) | .263594. |
| **30-39** | 22 (11) | 2 (2) | .000929. |
| **40-49** | 32 (16) | 8 (6) | .005332. |
| **50-59** | 36 (18) | 26 (20) | .76295. |
| **60-69** | 51 (26) | 31 (23) | .603024. |
| **70-80** | 36 (19) | 36 (27) | .056029. |
| **>80** | 12 (6) | 27 (20) | .000084. |
| **Days of enrolment after onset of symptoms, median (range)** | 5 | 6 | .94868. |
| **N° (%) of healthcare workers** | 32/128 (25%) | 17/128 (13%) | .0172 |
| **N° (%) of subjects with contact with sus-pected or confirmed COVID-19 case** | 82(42%) | 42(32%) | .0665 |
| **Charlson comorbidity index, median (IQR)** | 2 (3) | 3 (3) | .009547. |
| **N° (%) of subjects with underlying chronic disease** | 103/194 (53) | 94/130 (71) | .000516. |
| **With hypertension** | 78/172 (40) | 62/117 (47) | .201914. |
| **With cardio-vascular disease** | 41/180 (21) | 35/120 (27) | .212592. |
| **With diabetes** | 29/182 (15) | 25/126 (18) | .375271. |
| **With malignancy** | 16/183 (8) | 15/120 (12) | .291268. |
| **With chronic kidney disease** | 13/174 (7) | 14/118 (11) | .203487 |
| **With chronic obstructive pulmonary disease** | 23/182 (12) | 26/120 (20) | .037279. |
| **With liver cirrhosis** | 3/152 (2) | 3/116 (2) | .737002. |
| **N° (%) of subjects with** | | | |
| **Fever** | 126/138 (64) | 47/62 (36) | .003014. |
| **Cough** | 74/140 (38) | 32/62 (26) | .870263. |
| **Dyspnea** | 50/140 (25) | 32/62 (26) | .033816. |
| **hypo ageusia** | 15/102 (8) | 20/54 (15) | .001468. |
| **hyposmia** | 48/109 (24) | 23/56 (17) | .715658. |
| **Diarrhea** | 15/126 (8) | 8/59 (6) | .750579. |
| **N° (%) of subjects who died** | 6(3) | 53 (40) | .0000 |
